# Multifunctional role of DEAD-box helicase 41 in innate immunity, hematopoiesis and disease

**DOI:** 10.3389/fimmu.2024.1451705

**Published:** 2024-08-09

**Authors:** Jing Ma, Susan R. Ross

**Affiliations:** Department of Microbiology and Immunology, University of Illinois at Chicago College of Medicine, Chicago, IL, United States

**Keywords:** DEAD box helicase, innate immunity, AML/MDS, genomic stability, splicing

## Abstract

DEAD-box helicases are multifunctional proteins participating in many aspects of cellular RNA metabolism. DEAD-box helicase 41 (DDX41) in particular has pivotal roles in innate immune sensing and hematopoietic homeostasis. DDX41 recognizes foreign or self-nucleic acids generated during microbial infection, thereby initiating anti-pathogen responses. DDX41 also binds to RNA (R)-loops, structures consisting of DNA/RNA hybrids and a displaced strand of DNA that occur during transcription, thereby maintaining genome stability by preventing their accumulation. DDX41 deficiency leads to increased R-loop levels, resulting in inflammatory responses that likely influence hematopoietic stem and progenitor cell production and development. Beyond nucleic acid binding, DDX41 associates with proteins involved in RNA splicing as well as cellular proteins involved in innate immunity. DDX41 is also a tumor suppressor in familial and sporadic myelodysplastic syndrome/acute myelogenous leukemia (MDS/AML). In the present review, we summarize the functions of DDX helicases in critical biological processes, particularly focusing on DDX41’s association with cellular molecules and the mechanisms underlying its roles in innate immunity, hematopoiesis and the development of myeloid malignancies.

## 1 Introduction

**Figure 1:**
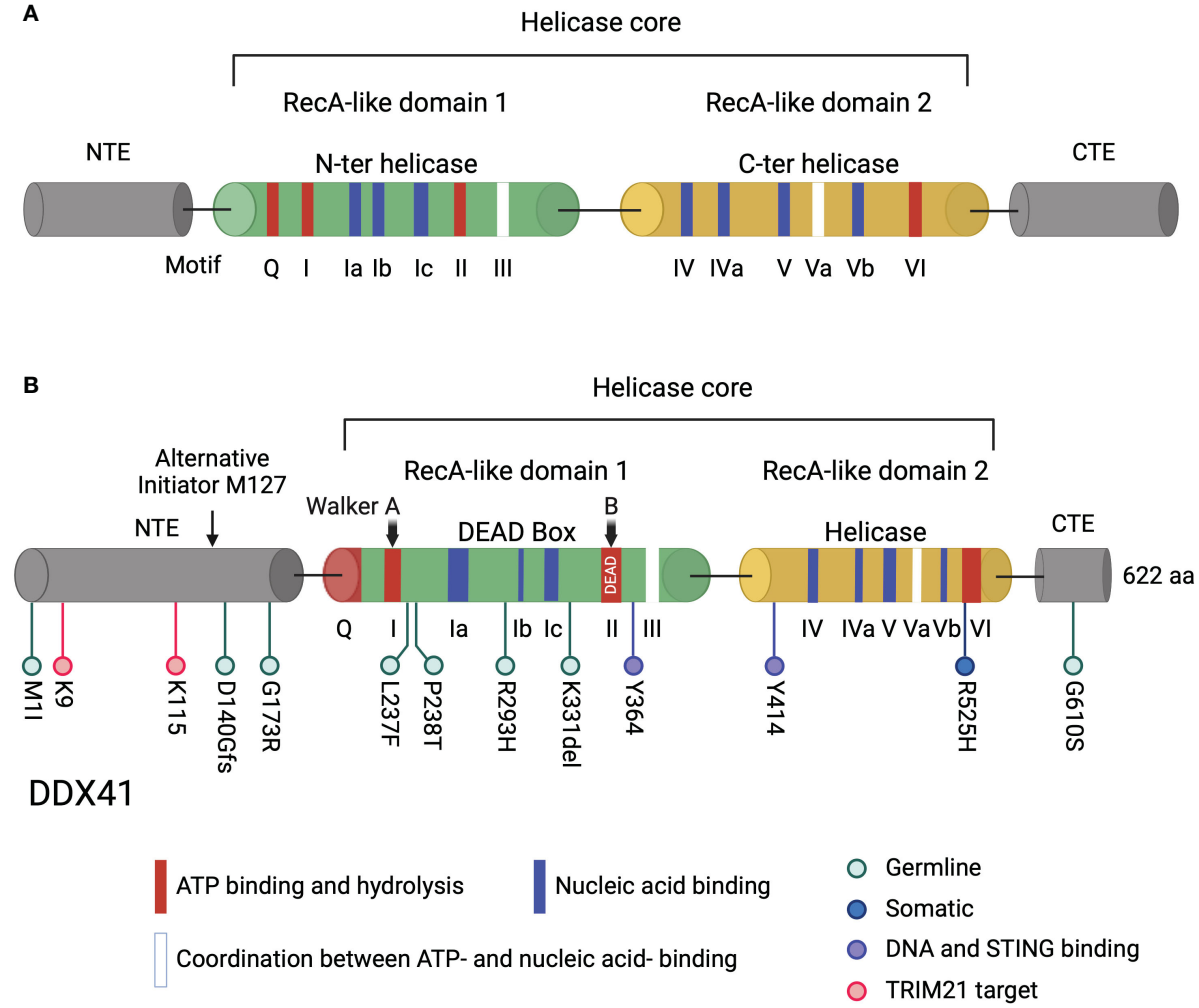
Domain structure of DDX helicases and DDX41. **(A)** Typical DDX helicases contain two conserved core domains, RecA-like domain 1 and RecA-like domain 2. Two RecA-like domains harbor motifs essential for ATP binding and hydrolysis (red), for nucleic acid binding (blue), and for coordination between ATP- and nucleic acid binding (white). The helicase core is flanked by an NTE and a CTE. **(B)** The long isoform of human DDX41 contains 622 amino acids. The helicase core is comprised of DEAD and helicase domains essential for ATP- and nucleic acid-binding. The Walker A and B motifs in the DDX41 DEAD domain, corresponding to motif I and II, are indicated. Walker B motif contains the amino acid sequence D-E-A-D. Germline and somatic DDX41 mutation sites in myeloid neoplasms are shown in light blue and dark blue, respectively, tyrosine sites critical for DNA and STING binding are shown in violet and TRIM21 target lysines are shown in pink below the diagram. The alternative translation initiation site M127 located in the NTE is indicated by an arrow. Created with BioRender.com.

RNA helicases are a large class of enzymes that play critical roles in RNA metabolism. They are classified into helicase superfamily 1 (SF1) and SF2. Within SF2, DEAD-box (DDX) helicases, which all contain a characteristic Asp-Glu-Ala-Asp (DEAD) motif, participate in cellular processes such as transcription regulation, pre-mRNA splicing, ribosome assembly, translation, RNA decay and innate immunity (1). Structurally, DDX helicases contain a conserved helicase core comprised of two DNA recombination and repair protein A (RecA)-like domains with motifs essential for ATP binding or hydrolysis, nucleic acid binding, and coupled binding of nucleic acid and ATP (1, 2) (**Figure 1A**). Typically, the helicase core is flanked by variable N-terminal (NTE) and C-terminal (CTE) extensions, which determine the protein’s specific targets (3) (**Figure 1A**). The human genome encodes 42 DDX helicases, 37 of which share conserved helicase motif structures, while 5 show divergence in these structures (4). The motif signatures of DDX helicases sets the foundation for their interaction with other molecules, thereby determining their function in different cellular processes. Sequence conservation highlights residues that are functionally important. Changes in the composition of even a few amino acids can create distinct isoforms, leading to altered or even loss of protein function. Dysregulation of DDX helicase expression frequently leads to cellular dysfunction and disease.

One DDX protein with diverse functions is DDX41. DDX41 was first studied because of its recognition of DNA or DNA/RNA hybrids generated during infection, triggering anti-viral and -bacteria immune responses (5–8). Phylogenetic analysis of DDX41 nucleic acid and amino acid sequences among mammalian and non-mammalian species indicates its evolutionary conservation in innate immunity (9–15). For example, fish DDX41 was found to have helicase domains similar to those in human DDX41, setting the foundation for testing functional conservation (16) (see below).

Among the well-studied helicases, DDX41 also stands out for its role in tumorigenesis. Cohort studies in MDS/AML identified DDX41 germline and somatic lesions, indicating that DDX41 is a tumor suppressor gene in myeloid malignancies (17). Germline DDX41 mutations, predominantly frameshift mutations, lead to haploinsufficient DDX41 expression due to loss-of-function (LOF) alleles (18–20). Subsequently acquired somatic DDX41 mutations of the unmutated allele enhance progression toward MDS/AML. Moreover, deletions of chromosome 5q (del(5q)) involving the DDX41 locus have been detected in patients with myeloid neoplasms (MN). Such deletions also result in haploinsufficient DDX41 expression and enhance progression to MDS and AML (18, 21, 22).

Screening for pathogenic variants in *DDX41* holds great clinical significance due to its critical role in disease progression. Germline variants of *DDX41* are the most prevalent mutations predisposing adults to myeloid neoplasms (23–25). Analysis of different subtypes of myeloid neoplasms (MNs) reveals the highest frequency of *DDX41* germline variants in secondary AML and high-risk MDS, followed by low-risk MDS, primary AML and MPN. Notably, patients with germline DDX41 mutations are characterized by long latency and favorable responses to treatment regimens (26–28).

Another aspect of DDX41 role is its role in hematopoiesis. DDX41 has been proposed to regulate hematopoiesis by multiple different mechanisms: DDX41 regulates gene expression and is required in stem cell differentiation and expansion (29, 30); DDX41 associates with spliceosome proteins and is involved in pre-mRNA splicing (18, 30); DDX41 plays a crucial role in maintaining R-loop homeostasis thereby affecting inflammatory signaling and genome stability (31–33), and DDX41 regulates snoRNA processing such that DDX41 LOF leads to defects in ribosome biogenesis (34–36).

## 2 Cellular functions of DEAD-box RNA helicases

### 2.1 Genome stability

DEAD-box helicases play a pivotal roles in maintaining genome stability. These helicases, which have high conservation of the core domain responsible for substrate binding and unwinding, are involved in recognizing cellular nucleic acids and proteins. DEAD-box helicases are thought to protect genome stability through this dual specificity. For instance, DDX1 is recruited to DNA breaks to remove DNA/RNA hybrids, thereby allowing repair to proceed (37–39). Similarly, DDX1 associates with the nuclear RNA exosome, and this interaction is sensitive to DNA damage (40). The RNA exosome targets aberrant transcripts produced upon DNA damage and processes/degrades RNAs by its ribonuclease activity (41). DDX1 acts as a cofactor in this process, facilitating RNA exosome function by its helicase/RNA binding activities (42, 43)). Moreover, DDX1 promotes DNA/RNA hybrid formation during antigen receptor recombination (44). DDX1 binding to G-quadruplexes (G4) facilitates their resolution and R-loop formation, resulting in the promotion of class switching recombination.

In contrast, DDX5 plays a role in limiting DNA/RNA hybrid accumulation by resolving DNA/RNA hybrids, enabling proper DNA repair at nearby double-strand breaks (DSBs) (45). DDX5’s association with RNA is dependent on its helicase activity, and it interacts with the DNA repair protein BRCA1, stimulating its unwinding activity and facilitating DNA repair by homologous recombination (45). DDX39B facilitates DNA repair by upregulating BRCA1 expression (46) and rescuing DNA damage mediated by R-loops (47). DDX21 shields cells from DNA damage and genome instability by limiting the level of DNA/RNA hybrids (48). It has also been suggested that *DDX41* LOF mutations induce R-loop-dependent DNA replication stress and genome instability (29, 32, 49). However, the molecular mechanisms underlying DDX41’s role in maintaining genome stability are still under exploration (see below).

These findings underscore the crucial function of DEAD-box helicases in safeguarding genome stability by regulating DNA/RNA hybrid levels. Depleting individual DDX proteins causes DNA damage, which suggests that these helicases plays distinct, non-redundant roles in promoting genome stability (38, 46, 48, 50–53).

### 2.2 Splicing alteration and tumorigenesis

Apart from their role in regulating nuclear RNA involved in genome maintenance, DDX helicases engage with splicing factors, potentially modulating splicing of cellular proteins. For example, both DDX42 and DDX46 interact with SF3B1, a core component of the human spliceosome U2 small nuclear ribonucleoprotein (snRNP). Disrupted binding to SF3B1 may lead to impaired assembly of the pre-spliceosome. Structural analysis reveals that DDX42 and DDX46 bind to SF3B1 residues that are commonly mutated in cancer, including the most frequently mutated residue K700 (54). Similarly, Zhao et al. found that a K700E mutation in SF3B1 disrupts its interaction with DDX42. Notably, overexpression of wild-type (WT) DDX42 restored this interaction, rescuing the aberrant splicing patterns induced by these mutations (55).

Recent studies have revealed DDX41’s protein interaction network, showing its association with spliceosome U2 and U5 snRNPs. Mutations in DDX41 linked to AML/MDS disrupt these interactions, leading to aberrant splicing patterns (18). Similarly, investigations into DDX3X have highlighted its involvement in splicing defects linked to cancer: DDX3X interacts with splicing factors, modulating alternative splicing of cancer-related genes and facilitating breast cancer adaptation to hypoxia and nutrient deprivation (56). Although DDX3X has been implicated in breast cancer development, contradictory evidence suggests its role as a tumor suppressor. Specific deletion of DDX3X in hepatocytes resulted in the development of liver tumors, accompanied by disruptions in DSB repair pathways and compromised genome integrity (57). Additionally, DDX3X appears to regulate the proliferation and migration of melanoma cells by translational repression of the oncogene MITF. DDX3X LOF is also associated with melanoma progression (58).

Taken together, these data suggest that DDX helicases are intricately associated with splicing machinery and may influence alternative RNA splicing in the context of cancer-related mutations. Additionally, since DEAD-box helicases play critical roles in key drivers of cancer, such as genome instability, cell cycle dysregulation, aberrant cell growth, and programmed cell death, it is not hard to imagine their involvement in cancer progression (59, 60).

### 2.3 Pro- or anti-viral activities of DDX helicases

DDX helicases also function in pathogen recognition and replication by interacting with either viral genomes or cellular factors. DDX60 is an antiviral factor that either promotes innate immunity or directly targets viral components (61, 62). In Vesicular Stomatitis Virus (VSV)- or Sendai Virus-infected cells, DDX60 promotes retinoic acid-inducible gene-I (RIG-I)-mediated type I interferon (IFN) expression. RIG-I, an intracellular sensor of viral RNA, activates downstream antiviral responses. DDX60 interacts with RIG-I, and co-expression of DDX60 and RIG-I increased the binding of RIG-I and DDX60 to dsRNA (61). Moreover, DDX60 exhibits RIG-I-independent antiviral activity by promoting degradation of VSV and Hepatitis C Virus RNA. DDX60 was found to interact with core components of the RNA exosome, including EXOSC1 and EXOSC4, leading to viral RNA degradation (62). Additionally, DDX60 reduces viral translation by decreasing ribosome occupancy on viral internal ribosome entry sites (IRESs). DDX60 selectively targets type II IRES RNA, including that of encephalomyocarditis virus and foot and mouth disease virus, causing a net reduction in virus translation (63).

Although DEAD-box helicases play an important role in anti-viral innate immunity, viruses often exploit them to evade immune responses and enhance their replication. For instance, DDX5 promotes replication of the RNA virus Japanese encephalitis virus by binding to its 3’ untranslated repeat (UTR); this action depends on the helicase activity (64). Conversely, DDX5 dampens innate immune responses to VSV infection by regulating viral RNA methylation (65). RNA methylation is a post-transcriptional modification involving transfer of a methyl group, and one of the most common RNA methylation modifications is N6-methyladenosine (m^6^A) (66). m^6^A methylation accounts for important regulatory mechanisms in gene expression and a diversity of physiological processes. DDX5’s P68HR domain, the unique tail found C-terminal to the helicase core, interacts with the RNA m^6^A methylase METTL3 and blocks methylation and nuclear export of host antiviral transcripts, such as the type I IFN and IL-6 mRNAs (65). Similar to DDX5, DDX46 promotes VSV infection by negatively regulating antiviral innate responses. Exclusively located in the nucleus, DDX46 recruits the RNA demethylase ALKBH5 to erase m^6^A modifications of antiviral gene transcripts, thus inducing their nuclear retention and reducing their translation (67).

## 3 DDX41 function in innate immunity

**Figure 2:**
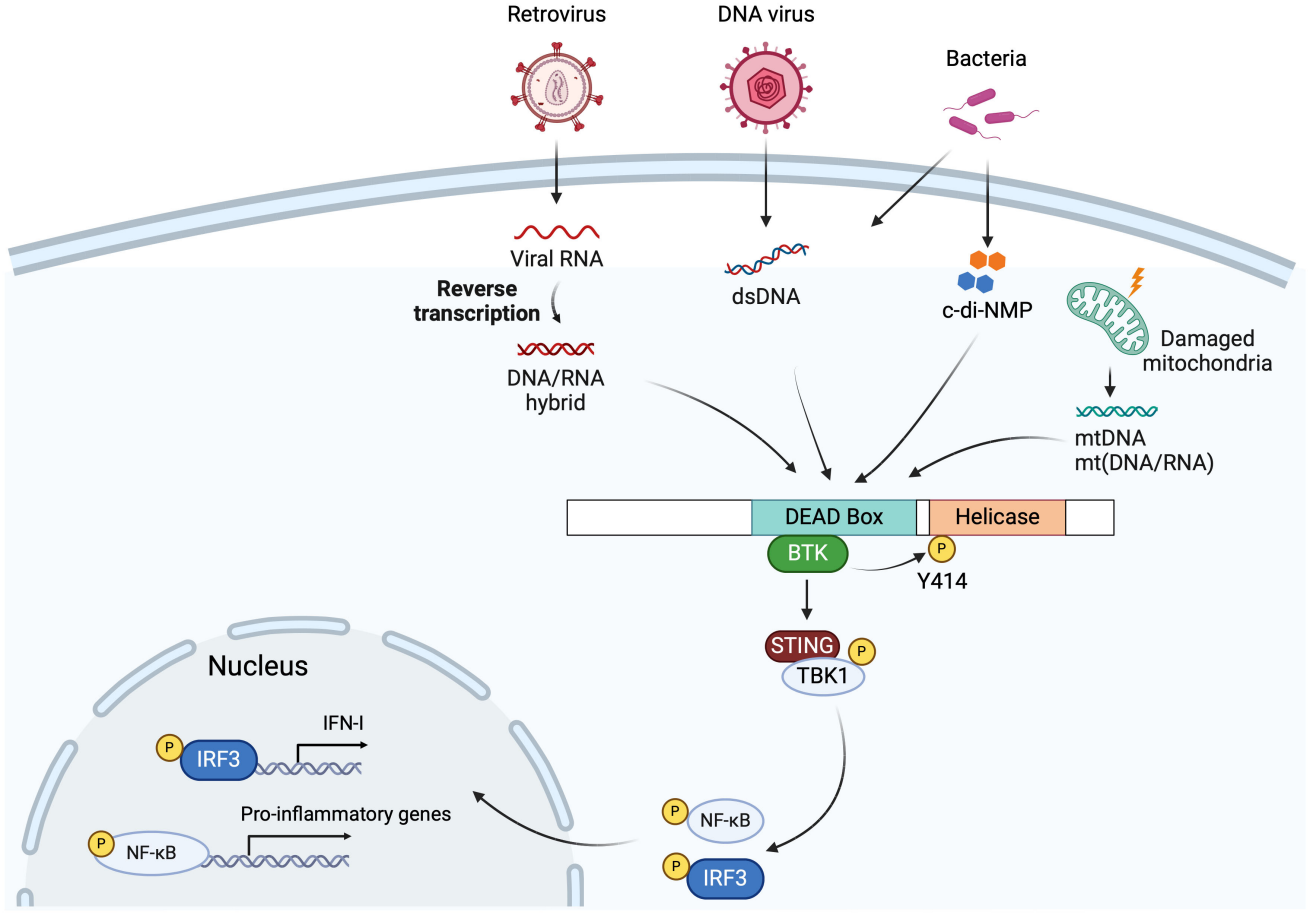
DDX41 in innate immunity. DDX41 recognizes cytosolic DNA or DNA/RNA hybrids generated during DNA virus and retrovirus infection via its DEAD domain, followed by BTK binding the DEAD domain and phosphorylating Tyr414. This facilitates DDX41 binding to STING, triggering the STING-TBK1-IRF3/NF-kB-type I IFN signaling pathway. Bacterial DNA or cyclic di-nucleotides (c-di-NMP), mitochondria DNA (mtDNA) and mitochondria DNA/RNA hybrids (mt(DNA/RNA)) generated by damaged mitochondria, are also sensed by the DDX41 DEAD domain, leading to STING pathway activation and production of type I IFNs and proinflammatory cytokines. Created with BioRender.com.

DDX41 is also involved in innate immune sensing. DDX41 was initially identified as a DNA sensor in a small interfering RNA (siRNA) screen targeting 59 members of the DEAD box and related DExD/H helicases (68). DDX41 recognizes cytosolic DNA originating from various sources and activates the Stimulator of IFN Genes (STING)-TBK1-IRF3-type I IFN signaling pathway (68, 69) (**Figure 2**). STING is typically activated when cyclic GMP-AMP synthase (cGAS) binds to double-stranded DNA (dsDNA), and synthesizes 2’3’ cyclic GMP-AMP (cGAMP), which binds to STING and activates it (70).

As a typical DDX helicase, the DDX41 helicase core is comprised of two conserved RecA-like domains, flanked by a NTE and a CTE (**Figure 1B**). The two core domains, DEAD and helicase, harbor conserved sequence motifs involved in ATP-binding and hydrolysis, in nucleic acid binding, and in the coordination of ATP and nucleic acid binding sites (2, 71, 72) (**Figure 1B**). Only the DEAD box domain is essential for DDX41 sensing of nucleic acids and cyclic dinucleotides (68, 72, 73). Motif I (Walker A) and motif II (Walker B) are conserved in all helicase superfamilies, and both Walker motifs in DDX41 are required for binding DNA and DNA/RNA hybrids (68, 74) (**Figure 1B**). After DDX41 binds nucleic acid, Bruton’s tyrosine kinase (BTK) binds the DEAD domain and phosphorylates Tyr414 (75). BTK activity facilitates DDX41 binding to STING and is required for STING-mediated type I IFN responses. Co-IP experiments showed that Tyr414 and another tyrosine residue, Tyr364, are required for DNA and STING binding (75) (**Figure 1B**).

In a mouse dendritic cell line, DDX41 depletion led to reduced IFN production in response to poly (dA:dT) and HSV-1 infection, but not to poly (I:C) (**Figure 2**) (68). DDX41 also triggers antiviral responses after infection with adenovirus, a DNA virus that replicates in the nucleus (76). In the mouse macrophage cell line RAW 264.7, DDX41 recognized endosome-escaped adenovirus DNA in the cell cytosol, a likely by-product of defective adenovirus particles, triggering IRF3 activation and subsequent type I IFN responses via the STING/TBK1/IRF3 pathway; knockdown of DDX41 in these cells diminished both IRF3 and STAT1/2 activation (77). In contrast, the same group showed that in the mouse endothelial cell line MS1, activation of both IRF3 and STAT1/2 occurred upon DDX41 knockdown during adenovirus infection, even though high levels of STING were detected in both cell lines (78). These data indicate cell-specific regulation of nucleic acid sensing during antiviral responses may occur.

Our lab also carried out an siRNA screen in a mouse macrophage cell line, to identify nucleic acid sensors that respond to retroviruses (79). One of the sensors identified was DDX41. We went on to show that during infection with retroviruses like murine leukemia virus (MLV) and human immunodeficiency virus (HIV), DDX41 primarily senses the DNA/RNA hybrid generated during the initial step of viral reverse transcription, particularly in dendritic cells, the initial targets of MLV infection, and that this then activated the STING pathway (5) (**Figure 2**). Upon virus infection, DDX41 binds STING, leading to phosphorylation of TBK1 and IRF3, and the production of type I IFN. Furthermore, mice with targeted knockout of DDX41 in dendritic cells were more highly infected with MLV than WT mice, confirming its anti-viral activity *in vivo*. Both *in vitro* and *in vivo* experiments showed that DDX41 functioned independently of cGAS to activate STING.

Recent studies also suggest that DDX41 can sense DNA/RNA hybrids released from damaged mitochondria during infection by other RNA viruses (6) (**Figure 2**). Influenza A virus infection causes mitochondrial damage and DDX41 recognizes mitochondrial DNA (mtDNA) and DNA/RNA hybrids released into the cytosol and triggers antiviral immune responses (6, 80). Independent of infection, in a model of misfolded mutant Superoxide dismutase (SOD1)-driven amyotrophic lateral sclerosis (ALS), damaged mitochondria-released mtDNA and mt(DNA/RNA) hybrids activated the cGAS-STING and DDX41-STING pathways, respectively (81). Additionally, DDX41 sensed R-loops, DNA/RNA hybrid structures, and triggered type I IFN responses and ISG (ISG15 and CXCL10) production in Myocyte-specific enhancer factor 2A (MEF2A)-depleted myocytes. MEF2A is a transcription factor potentially regulating inflammation (82).

In addition to its role in sensing viral nucleic acids, DDX41 triggers IFN responses during bacterial infections (**Figure 2**). Bacterial cyclic dinucleotides (CDNs), including the secondary messengers cyclic di-GMP (c-di-GMP) or cyclic di-AMP (c-di-AMP), act as PAMPs which are directly sensed by DDX41 in both human and mouse cells. Upon activation, DDX41 triggers the activation of the STING pathway (73) (**Figure 2**).

In non-mammalian species, Ddx41 also plays a critical role in innate immune signaling and the response to viral infection and DNA or RNA stimuli. Sequence analysis reveals high homology in Ddx41 among fish species (14, 16, 83–85). The crystal structure of zebrafish Ddx41 revealed a binding surface for both dsDNA and CDNs in the DEAD domain and highlighted a critical tyrosine phosphorylation site at Tyr405. This site corresponds to Tyr414 in human DDX41, mutation of which abrogates nucleic acid binding, although whether this is also the case for Tyr405 has not been tested (84). A study of Mandarin fish Ddx41 showed that both the DEAD and helicase domains interact with STING, while, similar to mammalian DDX41, only the DEAD domain is responsible for dsDNA binding (14).

Overexpression of *ddx41* in a mandarin fish cell line attenuated DNA or RNA virus infection, accompanied by cytokine production; activation of the type I IFN and NF-κB promoters was also observed in reporter assays (14, 85). Two studies showed that co-expression of *ddx41* and *sting* in fish cells challenged by DNA or a RNA virus had synergic effects on STING signaling compared to over-expression of *sting* only (14, 16). Moreover, reporter assays showed that after DNA stimulation, co-microinjection of *ddx41* and *sting* expression vectors in zebrafish embryos enhanced activation of the IRF3, NF-κB and IFN-I promoters compared to that single expression vectors (84). Type I IFN and IFN-stimulated gene (ISG) expression was also increased upon RNA stimulation by *ddx41* and *sting* expression vector-co-microinjection (84). In mammals, in addition to IRF3 and NF-κB, activated STING also activates signal transducer and activator of transcription 6 (STAT6), inducing expression of chemokines such as CCL20 (86, 87). In zebrafish, Stat6 was required for CCL20 production upon DNA stimulation, suggesting a similar signaling pathway (84). Collectively, these data indicate that fish Ddx41 senses DNA, RNA or viral infection, thereby activating STING-dependent immune responses.

Following stimulation with DNA or DNA virus infection, fish cell lines and tissues exhibited increased Ddx41 expression (14, 16, 83). In addition, bacterial infection, or lipopolysaccharide (LPS) treatment induced expression of *ddx41* in peripheral blood cells and spleen tissue of Clark’s anemonefish. One possibility is that LPS triggers mitochondrial damage, leading to mtDNA release into the cytosol, thereby inducing *ddx41* expression (85).

In contrast, *DDX41* is not IFN-inducible in mammals, and its expression is not induced by viral infection or LPS in mammalian cells (5, 6, 68). It is possible that the fish DNA sensing system differs from that in mammals (88). Ddx41-Sting-mediated signaling has not yet been fully demonstrated *in vivo* in fish (14, 16, 84).

How DDX41 binding triggers STING activation in mammalian cells is not yet fully understood. STING is known to be activated by cGAMP which is synthesized by cGAS upon binding dsDNA (89). One set of experiments suggested that upon DNA stimulation, DDX41 interacts with cGAS and produces dsDNA through DDX41’s strand-annealing activity, thereby activating cGAS and increasing 2’3’-cGAMP levels. When no stimuli exist, DDX41’s unwinding activity took over and produced ssDNA, thus inactivating cGAS-STING-IFN pathway (8). However, other research has suggested that it is DDX41 direct binding to STING activates it and that this pathway functions independently of cGAS (5, 6, 75). Moreover, DDX41 has not been shown to act through cGAS in fish during RNA sensing (90, 91).

DDX41-mediated responses can be abrogated by protein degradation. During DNA virus infection or DNA stimulation, the E3 ligase TRIM21 negatively regulates expression of DDX41 protein by binding to its DEAD domain. TRIM21 targets Lys9 and Lys115 of DDX41, leading to K48 ubiquitination, targeting DDX41 protein for degradation (92). This process likely exists as a means of dampening or turning off the innate immune response.

## 4 DDX41 in hematopoiesis and myeloid dysplasia

### 4.1 The role of DDX41 in hematopoiesis

**Figure 3:**
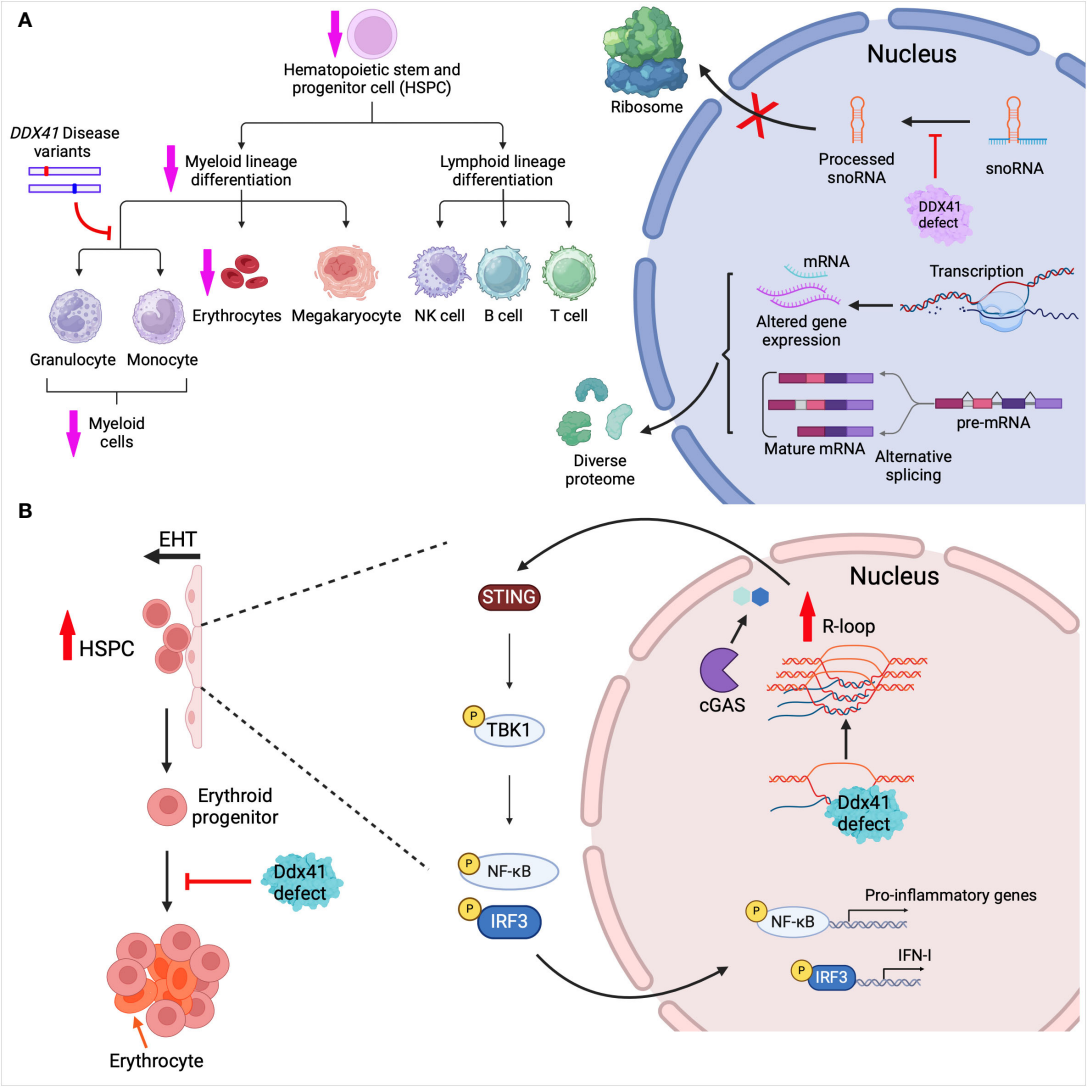
DDX41 in hematopoiesis. **(A)** *Ddx41* depletion in mouse hematopoietic system leads to myeloid differentiation defects and reduced erythrocyte levels. Mouse embryonic HSPCs lacking *Ddx41* showed differential gene expression and mRNA alternative splicing (AS). Mouse myeloid progenitor cell lines harboring pathogenic mutations of *Ddx41* had disrupted granulocytic/monocytic differentiation. AS-generated mRNAs and gene expression changes contribute to diverse protein isoforms. BM cells from adult DDX41-deficient mice are defective in snoRNA processing, leading to reduced ribosome biogenesis. **(B)** In zebrafish, HSPCs are produced via endothelial-to-hematopoietic transition (EHT). *Ddx41* defects in embryos lead to R-loop accumulation which promotes HSPC production. The aberrant accumulation of R-loops induces activation of the cGAS-STING pathway and production of type I interferons. During erythropoiesis, *Ddx41* defects also lead to improper erythrocyte expansion and maturation. Created with BioRender.com.

We showed that *Ddx41* germline knockout (KO) in mice led to early embryonic lethality, highlighting a role for *Ddx41* in development (5). In contrast, *Ddx41* KO in dendritic cells or macrophages had no impact on cell development or survival, and only affected anti-viral innate immunity. When we depleted *Ddx41* in mouse hematopoietic stem cells using the VavCre-loxP system and a floxed allele of *Ddx41*, the mice were born with hematopoietic defects and died within a few days of birth (30). *Ddx41* depletion caused anemia and a reduction in common myeloid progenitor cells (CMPs) in mouse embryos, whereas megakaryocyte–erythroid progenitor cells (MEPs) or common lymphoid progenitor cells (CLPs) were not affected (**Figure 3A**). In BM transplant studies, adult mice that received cells carrying monoallelic *Ddx41* mutations had normal hematopoiesis, while those receiving biallelic mutations BM cells showed hematopoietic defects (30, 35). We performed whole transcriptome sequencing of embryonic mouse hematopoietic stem and progenitor cells (HSPCs). *Ddx41* depletion in HSPCs resulted in upregulation of genes involved in blood vessel development and vasculogenesis, and downregulation of genes enriched in inflammation, immune response, and leukocyte differentiation (**Figure 3A**). Differentially expressed transcription factors were overrepresented in Gene Ontology terms related to embryonic development and cell differentiation in KO verses WT HSPCs. Moreover, alternative splicing was also observed in *Ddx41*-depleted HSPCs. Further research is needed to elucidate the biological significance of DDX41-mediated gene differential expression and alterative splicing in hematopoietic stem cell differentiation.

Recent research has underscored the indispensable role of Ddx41 in erythrocytic development in zebrafish (**Figure 3B**). Zebrafish with a *ddx41* containing a premature stop codon at Tyr410, leading to the deletion of the helicase domain and C-terminal region, were used to study its role in erythropoiesis (29). Ddx41 was found to play a crucial role in preventing genomic stress and promoting proper erythropoiesis, particularly in the expansion of erythroid progenitors (**Figure 3B**). Transcriptome analysis revealed both mis-expression and alternative splicing in genes associated with the cell cycle in *ddx41* mutant erythroid progenitors. Further analysis indicated that Ddx41-regulated Ataxia-telangiectasia-mutated (ATM) and -Ataxia-telangiectasia and Rad3-related (ATR) expression were critical for normal expansion of erythroid progenitors.

As mentioned above, DDX41 binds both DNA and DNA/RNA hybrids, contributing to innate immune signaling. R-loops, formed during transcription when the nascent RNA anneals with the template DNA strand, comprise a DNA/RNA hybrid and a displaced single strand DNA. R-loops have the potential to disrupt DNA replication, DNA repair and transcription, posing a threat to genome integrity and function (80). DDX41 possesses DNA-RNA unwinding activity and consequently, DDX41 has been implicated in both normal hematopoiesis and AML/MDS in preventing the deleterious effects of pathological R loops. Weinreb et al. were the first to report a role for Ddx41 in constraining R-loop levels during zebrafish hematopoiesis (**Figure 3B**) (31). As described above, they established a hypomorphic *ddx41* mutant with deletion of the helicase domain (**Figure 1B**), with greatly diminished but not completely absent expression. Expression of this hypomorphic *ddx41* led to the aberrant accumulation of R-loops, which, in turn, promoted a substantial increase in the production of zebrafish HSPCs. The disrupted R-loop homeostasis in the *ddx41* mutant resulted in higher inflammatory signaling that depended on activation of the cGAS-STING pathway and elevation of type I IFNs and their target genes in the *ddx41* mutants (**Figure 3B**). This suggests that zebrafish Ddx41 negatively regulates R-loop formation and restricts inflammatory signaling during hematopoiesis, highlighting its importance for HSPC maintenance. Interestingly, this association between inflammation and hematopoiesis in *ddx41*-depleted cells extends from zebrafish to humans. Notably, Reactome Pathway analysis highlighted the immune response pathway in *DDX41*-low CD34^+^ HSPCs from MDS patients; there was a strong correlation between upregulation of genes involved in innate immune response and high counts of BM blasts (31).

### 4.2 DDX41’s role in genomic stability and transcriptional regulation

In addition to its role in inflammation, it has been shown that in K562 leukemic cells, *DDX41* deficiency leads to accumulation of R-loops and DNA replication defects (32). Unlike splicing factors that tend to favor exon inclusion or exclusion, *DDX41*-deficient cells did not have a particular bias toward any specific alternative splicing event (32). In this model, DDX41 binding to 5’ splice sites (SS) increased R-loop formation, thereby stalling RNA polymerase II and creating a potential barrier to DNA replication (93, 94). However, inhibition of *DDX41* expression showed an anti-proliferative effect in K562 cells, which is the opposite to what Polprasert et al. observed in the same cell line (18). Unrepaired DNA damage persisted through mitosis, gradually accumulating in aged HSCs, priming cells into a premalignant state, a phenomenon frequently observed in the bone marrow of patients with *DDX41* mutations (32, 95).

In line with Shinriki et al.’s findings, Mosler et al. proposed a model in which the absence of *DDX41* impedes the progression of replication forks, triggering replication stress response (49). Differing from the observations of Shinriki’s group, which suggested that DDX41 prevents R-loop accumulation primarily by influencing R-loop formation rather than resolution, Mosler and colleagues proposed that DDX41 directly bound to and unwound DNA/RNA hybrids at the promoters of active genes. In their study, over-expression of *DDX41* carrying pathogenic mutations in either the DEAD (L237F/P238T) or helicase (R525H) domains (**Figure 1B**) led to reduced efficiency in unwinding R-loops in an *in vitro* assay. Additionally, the enforced expression of pathogenic variants of *DDX41* resulted in the accumulation of DNA damage in *DDX41*-deficient human HSPCs. Thus, these findings suggest that DDX41 functions in genome integrity as well as in RNA splicing. Future advancements in this area may determine how preventing R-loops formation promotes hematopoietic differentiation and proliferation by DDX41, as well as the effect of specific mutations on this process.

### 4.3 Impact of *DDX41* mutations on ribosome biogenesis and snoRNA procession in disease pathogenesis

It has also been suggested that DDX41 affects hematopoiesis and myeloid neoplasia through altering ribosome function (34, 35). Over-expression of *DDX41* R525H in human hematopoietic cells caused a ribosomopathy phenotype (34). The mutant DDX41 retained RNA-binding activity but exhibited lower ATPase activity, functioning in a dominant-negative manner (34). In human cord blood derived CD34^+^ cells, overexpression of *DDX41* R525H also led to growth inhibition. Gene set enrichment analysis revealed downregulation of ribosomal genes in cells expressing *DDX41* R525H; cell cycle genes regulated by the retinoblastoma (RB) – eukaryotic transcription factor (E2F) axis were down-regulated as well, suggesting that cell cycle inhibition by this mutant is RB-dependent. RB, a key regulator and main binding partner of E2F, is linked to abnormal proliferation and genomic errors generated during the cell cycle (96). Whether both functions are required for the mutant DDX41’s effects on hematopoiesis is unclear.

Chlon and colleagues also found small nucleolar RNA (snoRNA) processing defects associated with the R525H mutation. They crossed *Ddx41* ^R525H^ conditional knock-in (KI) mice with *Ddx41^f^* ^loxed^ and Rosa26-CreERT2 mice, generating mice with combined LOF and R525H mutations upon tamoxifen-inducible Cre activation. Lineage-negative (Lin^-^) BM cells from *Ddx41* ^-/-^ or *Ddx41* ^KI/-^ mice were immortalized, then analyzed by deep sequencing: They found that *Ddx41* is required for snoRNA processing (35) (**Figure 3A**). snoRNAs are short, non-polyadenylated, non-coding RNAs that guide snRNPs to catalyze chemical modifications of ribosomal RNA (rRNA) and transfer RNA (tRNA) and are thereby important in ribosome biogenesis and protein synthesis (97) (**Figure 3A**). The mutant Ddx41 may have caused defects in processing of snoRNAs due to incomplete removal of introns from host genes.

Mice with monoallelic mutations *Ddx41* (+/- or R525H/+) in their HSCs did not exhibit hematopoietic defects, while those with biallelic mutations (-/- or R525H/-) showed impairment of hematopoiesis (**Figure 1B**) (35). Decreased abundance of mature snoRNA, reduced protein synthesis, and ribosome defects were detected in the cells harboring biallelic mutations (both -/- and R525H/-). What is noteworthy about the R525H mutation is that it is not a complete loss-of-function mutation, and the ribosome defects were less profound in Ddx41^R525H/-^ than in Ddx41^-/-^ bone marrow cells. The residual activity in the R525H mutant may allow mutant HSCs to persist because of the low protein translation demands in these cells. Furthermore, Chlon et al. observed dysregulation of snoRNA processing in the BM cells of *DDX41* mutant MDS patients. This suggests a snoRNA-related role of *DDX41* in MDS pathogenesis.

### 4.4 DDX41 interaction with proteins involved in RNA splicing

DDX41’s capacity to bind various nucleic acids (dsDNA, pre-mRNA, DNA/RNA hybrids and R-loops) may enable its involvement in numerous essential biological activities. However, it is clear that DDX41’s interaction with cellular proteins also plays a role in its disease-modifying function in myeloid neoplasms. To identify DDX41 interacting proteins, Polprasert and colleagues conducted mass spectrometry analysis of proteins bound to overexpressed human DDX41 (18). The interacting proteins were categorized into different functional groups, including heterogeneous nuclear ribonucleoproteins (hnRNP), NONO-SFPQ proteins, which are telomere repeat-associated proteins, and splicing-associated proteins/complexes, including spliceosomal, serine-arginine rich (SR) and exon junction complex proteins. Interestingly, mutations in spliceosome proteins are frequently found in myeloid neoplasms, but they appear to be mutually exclusive with *DDX41* mutations in MDS/AML patients (18). Additionally, the DDX41 R525H mutant exhibited reduced binding to these spliceosomal proteins, particularly the major components in U2 and U5. While splicing alterations have been detected with complete loss of DDX41, different splicing patterns with the R525H mutant have not yet been shown (30–32, 98).

Shinriki’s group also carried out MS analysis of DDX41-interacting proteins and found that it binds to NineTeen complex (NTC) (32). NTC components not only facilitate spliceosome assembly, but also play a role in transcriptional elongation. Aberrant splicing or paused transcription elongation has been linked to leukemogenesis in hematopoietic cells (99). By coordinating RNA splicing and transcriptional elongation, DDX41 may also help maintain genomic stability.

### 4.5 DDX41 mutations in myeloid neoplasms

Familial cases of AML/MDS cases have been extensively investigated, revealing both inherited and somatic mutations in *DDX41*. *DDX41* is one of the most highly mutated genes in familial AML/MDS; other genes include tet methylcytosine dioxygenase 2 (*TET2*), *RUNX1*, *GATA2* and the tumor suppressor gene *TP53*, which is mutated in many cancers (100–104). TET2 silences gene expression by methylation while RUNX1 and GATA2 are transcription factors important for hematopoietic stem cell differentiation (100–103). *DDX41* germline mutations typically result in frameshift alterations leading to loss-of-function, such as the mutation hotspot D140Gfs, while somatic mutations commonly involve missense alterations, resulting in hypomorphic proteins that may retain some function or may play a dominant role in carcinogenesis, such as the most commonly acquired mutation R525H (21, 25, 26, 35, 105) (**Figure 1B**). Complete loss of *Ddx41* is incompatible with hematopoietic stem cell viability and biallelic loss of function germline mutations of *DDX41* are rarely found in patient cohorts (30, 105, 106). Moreover, germline *DDX41* knockout in mice leads to early embryonic lethality (5).

The M1I mutation is one of the most prevalent germline mutations in the Caucasian population (18, 23, 24) (**Figure 1B**). Unlike the D140G mutation, M1I results in the loss of the methionine start codon and produces a truncated protein through use of an alternative translational initiation site (21, 34) (**Figure 1B**). Both isoforms of DDX41 have been detected in AML cell lines, and revealed changes in the subcellular localization of the truncated protein compared to WT DDX41; the truncated protein isoform is reduced in the nucleus and increased in the cytoplasm compared to the full-length isoform (19, 34). This observation raises the possibility that a decrease in the amount of nuclear DDX41 causes functional defects in DSB repair, transcription or splicing. In contrast, the R525H mutant retains residual DDX41 function and is compatible with HSC survival (35). Patients with myeloid neoplasms harboring either M1I germline or R525H somatic mutations did not exhibit differences in overall survival to other patients (21). Nevertheless, there may be a broader range of phenotypes associated with the M1I mutation, necessitating further investigation in larger cohorts (107).

To discriminate the activities of WT *Ddx41* and disease-associated variants of *Ddx41*, Kim et al. utilized a mouse hematopoietic progenitor cell line, which can be differentiated to neutrophils or macrophages, depending on the culture media (108). They studied the function of missense germline mutations G173R, R293H, G610S, missense somatic mutation R525H, and nonsense germline mutation K331del by transducing individual variants into *Ddx41* ^+/-^ progenitor cells (28) (**Figure 1B**). Despite maintaining nuclear localization similar to WT *Ddx41*, all the variants displayed attenuated transcript regulatory activity, with G173R and K331del being the most defective. G173R and K331del were also inactive in promoting granulocytic differentiation compared to WT, while R525H retained partial activity. The *Ddx41* disease variants may have disrupted the balance of monocytic and granulocytic lineage during myeloid differentiation (**Figure 3A**). This approach has the potential to uncover the structure/function relationship in patient derived *DDX41* mutations.

### 4.6 DDX41 in solid cancers

Aberrant DDX41 expression has been observed in solid tumors, but its precise function has yet to be determined (109, 110). Notably, higher levels of *DDX41* were found in many types of solid tumors; in hepatocellular carcinoma, elevated expression of *DDX41* correlated with increased tumor grade (111) while in clear cell renal cell carcinoma, increased *DDX41* was associated with tumor growth and poor prognosis (112). In breast cancer, lower patient survival rate was linked to higher expression of *DDX41* and endogenous *DDX41* in a breast cancer cell line promoted cell growth and colony forming capacity (113). Furthermore, pathogenic germline variants of *DDX41* have also been identified in solid cancers such as laryngeal, breast, and prostate cancer, either alone or concomitant with hematopoietic malignancies (23, 114, 115). These data indicate that DDX41 is an oncogene in many types of solid tumors.

However, a study in HeLa cells indicated an opposite role for DDX41 in tumorigenesis, as overexpression of DDX41 in these cells suppressed proliferation and induced apoptosis (116). RNA-seq analysis showed that expression of certain oncogenes were up- or down-regulated in response to DDX41 over-expression. Interestingly, the direction of regulation of these oncogenes (DDX41-overexpressing HeLa vs. WT HeLa) was consistent with their regulation in the cervical and endocervical squamous cancer (CESC) dataset (normal vs. cancer). This suggested a potential tumor suppressor role for DDX41 in some solid tumors. Moreover, the regulation of immune-associated genes in the DDX41-overexpressing HeLa cells correlated with findings from the CESC dataset analysis, indicating a potential link between DDX41 and tumor immunity.

## 5 Conclusions

DDX helicases family play multiple roles in different cellular processes. Among these, DDX41 in particular emerges as a versatile protein with multiple functions. It binds nucleic acids and proteins and participates in essential biological activities, such as innate immunity, genome integrity, RNA splicing and regulation of gene expression (5, 6, 68, 69, 73, 75, 80). The DDX41 core DEAD and helicase domains harbor important motifs and binding sites for sensing pathogen nucleic acids and initiating STING-mediated type I IFN responses (68, 74, 75). Although DDX41 recognizes cytosolic DNA and initiates the STING-TBK1-IRF3-IFN I signaling pathway, during infection with retroviruses such as MLV and HIV, DDX41 primarily senses the DNA/RNA hybrids generated during viral reverse transcription (5, 75). Furthermore, during Influenza A virus infection, DDX41 senses mtDNA and DNA/RNA hybrids released from damaged mitochondria, thereby eliciting antiviral immune responses (6, 80). Studies on fish Ddx41 suggest conservation in core domains between fish and mammalian DDX41, as well as similarities in triggering STING-mediated signaling (14, 16, 83–85).

Beyond its immune sensing functions, DDX41 has been identified as a tumor suppressor gene in myeloid neoplasms. Both inherited and sporadic mutations in *DDX41* have been identified in patients with myeloid neoplasms (18, 24, 25). In zebrafish models, *ddx41* hypomorphic mutations disrupt proper erythropoiesis, while in mouse embryos, hematopoietic depletion of *Ddx41* reduces myeloid progenitors without affecting erythroid progenitors (29, 30). Mechanistically, *ddx41* depletion leads to aberrant accumulation of R-loops, which is linked to elevated inflammation during hematopoiesis and DNA replication stress (31). Moreover, DDX41 lesions induced pre-mRNA splicing defects in both patient cells and mouse HSPCs (18, 30). Over-expression of mutant DDX41 R525H altered its interaction with major components of the U2 and U5 spliceosomes (18). In addition, DDX41 regulates snoRNA processing and ribosome biogenesis (35). Dysregulation of these processes by reduced function of DDX41 may contribute to MDS/AML.

Gene testing approaches assist in diagnosing myeloid neoplasias by identifying heterozygous germline pathogenic variants in *DDX41*, along with additional somatic variants from malignant myeloid cells (117). These approaches facilitate early diagnosis and clinical monitoring of individuals carrying familial *DDX41* pathogenic variants (118). Patients with *DDX41* pathogenic germline variants showed improved response to treatment and prolonged overall survival, including responses to the drug lenalidomide (18, 119), intensive chemotherapy (23, 120), and hypomethylating agents (24). However, donor-derived leukemia has been reported in allogeneic hematopoietic stem transplantation (HSCT) recipients when donors carry pathogenic germline *DDX41* variants (121–123). Therefore, it is beneficial to identify familial variants of *DDX41* to avoid unexpected outcomes of treatment. Research on *DDX41* sheds light on potential therapeutic target molecules and pathways in AML cell with *DDX41* mutations. For instance:

• *DDX41* deficiency-induced R-loop elevation leads to DNA damage, suggesting agents targeting DNA damage might impede disease progression (124).

• ATR, a protein promoting cell survival under DNA damage conditions, emerges as a promising therapeutic target due to AML cells dependency on ATR signaling in *DDX41* deficiency (49). Given that ATR activation is linked to DDX41-/STING-mediated IFN signaling in response to cellular stress, ATR kinase inhibitors may alleviate deleterious inflammation in neuroinflammatory and proliferative diseases (82).

• Loss of DDX41-induced inflammatory signaling contributes to hematopoietic expansion, indicating a potential therapeutic pathway for therapeutic intervention (125).

Although the mechanisms underlying DDX41’s tumor suppressor function have been elucidated to some extent, the structural and functional consequences of pathogenic variants of *DDX41* remain unclear. Moreover, how DDX41 and other cellular factors precisely control physiological R-loops has yet to be determined. Lastly, it is important to elucidate downstream regulators that mediate the global changes in pre-mRNA splicing, and to understand the dynamics of DDX41 interacting with the spliceosome.
